# *Propionibacterium acnes* Augments Antitumor, Anti-Angiogenesis and Immunomodulatory Effects of Melatonin on Breast Cancer Implanted in Mice

**DOI:** 10.1371/journal.pone.0124384

**Published:** 2015-04-28

**Authors:** Wamidh H. Talib, Suhair Saleh

**Affiliations:** 1 Department of Clinical Pharmacy and Therapeutics, Applied Science University, Amman, Jordan; 2 Department of Pharmaceutical Sciences and Pharmaceutics, Applied Science University, Amman, Jordan; University of Ulster, UNITED KINGDOM

## Abstract

Breast cancer is one of the most invasive cancers with high mortality. The immune stimulating *Propionibacterium acnes* is a Gram positive bacterium that has the ability to cause inflammation and activate Th1-type cytokine immune response. Antitumor response was associated with the inflammation induced by *P*. *acnes*, but the antitumor effect of this bacterium was not evaluated in combination with other agents. The aim of this study was to test the antitumor potential of a combination of melatonin and *P*. *acnes* against breast cancer implanted in mice. Balb/C mice were transplanted with EMT6/P cell line and *in vivo* antitumor effect was assessed for *P*. *acnes*, melatonin, and a combination of melatonin and *P*. *acnes*. Tumor and organs sections were examined using hematoxylin/eosin staining protocol, and TUNEL colorimetric assay was used to detect apoptosis. The expression of vascular endothelial growth factor (VEGF) was measured in tumor sections and serum levels of INF-γ, and IL-4 were measured to evaluate the immune system function. To evaluate the toxicity of our combination, AST and ALT levels were measured in the serum of treated mice. The combination of melatonin and *P*. *acnes* has high efficiency in targeting breast cancer in mice. Forty percent of treated mice were completely cured using this combination and the combination inhibited metastasis of cancer cells to other organs. The combination therapy reduced angiogenesis, exhibited no toxicity, induced apoptosis, and stimulates strong Th1-type cytokine antitumor immune response. The combination of melatonin and *P*. *acnes* represents a promising option to treat breast cancer. However, carful preclinical and clinical evaluation is needed before considering this combination for human therapy.

## Introduction

The high incidence and mortality of breast cancer made it a global health problem [1]. It is the most common type among women and characterized by high progression and metastasis [2]. Each year over a million new cases of breast cancer are diagnosed worldwide and the rates are in continuous rise [3]. Cancer progression depends on integrated steps including angiogenesis, metastasis, proliferation, and immune evasion [4]. In addition to the altered redox status, breast cancer is associated with excessive proliferation, unregulated differentiation, and low apoptosis rate [5]. Current therapeutic approaches (surgery, chemotherapy, and radiation) showed limited success to treat breast cancer [6]. Therefore, using combination therapies that target multiple steps in cancer development may produce more effective treatment.

Melatonin is a pineal gland hormone that has the capacity to inhibit tumor growth using direct and indirect mechanisms. It has pro-apoptotic, antiproliferative, anti-angiogenic, immune-stimulation, and free radical scavenging activities against different cancers [7]. It exerts its anticancer effects on breast cancer by inhibiting cell proliferation and angiogenesis [8] and exhibit more effectiveness against estrogen receptor positive breast cancer cells [9]. Although various studies demonstrated a clear anticancer effect of melatonin, it cannot alone cause a complete regression of a tumor [7]. However, melatonin can act synergistically (or additively) to potentiate the anticancer effects of other agents [10].

*Propionibacterium acnes* (*P*. *acnes*) is a Gram positive, anaerobic normal bacterial flora of the skin that is suggested to participate in the development of acne [11]. Early experiments on this bacterium showed effective antitumor activity against various animal and human cancers [12]. The anticancer effect of *P*. *acnes* involves different mechanisms including activation of NK cells [13], macrophages stimulation [14], and T-cell mediated anti-tumor immunity [15]. Additionally, inflammatory neutrophils were also suggested to participate in the anticancer response induced by *P*. *acnes* [16]. Th1-type cytokine immune response is associated with the phagocytosis of *P*. *acnes* by dendritic cells. This immune response causes an increase in the production of IFN-γ, TNF-α, and IL-12 [17]. The immune-modulatory effect of *P*. *acnes* encouraged scientist to use this bacterium to break cancer immune evasion. Accordingly, intratumoral injection of *P*. *acnes* causes tumor regression and an increase in monocytes and CD8^+^T lymphocytes that produce IFN-γ and TNF-α [18].

In spite of the different reports that explained the immune-modulatory activity of *P*. *acnes*, some studies showed a failure of this bacterium to induce antitumor immune response [19, 20]. Additionally, combinations of *P*. *acnes* with chemotherapy produced no improvement when tested on breast cancer patients [20, 21]. An explanation for the failure of these combination therapies is the fact that most anticancer chemotherapeutic agents induce immunosuppression which antagonizes the immune-modulation effect of *P*. *acnes*. Additionally, chemotherapeutic agents may inhibit *P*. *acnes* reducing its anticancer effect, Thus, combination of *P*. *acnes* with an agent that is less toxic and able to augment its immune-modulatory effect may produce better results.

In the current study, we have evaluated the potential of melatonin to augment the immune-modulatory effect of *P*. *acnes* and have hypothesized that a combination of melatonin and *P*. *acnes* may work synergistically (or additively) to inhibit breast cancer *in vivo* possibly by inhibiting angiogenesis, enhancing anticancer immune response, and inducing apoptosis.

## Materials and Methods

### Ethical statement

Animal care and use were conducted according to standard ethical guidelines, and all of the experimental protocols were approved by the Research and Ethical Committee at the Faculty of Pharmacy—Applied Science University.

### Experimental animals

Female Balb/C mice (6–8 weeks old, 23–25 g body weight) were used in this study. Separate cages with wooden shaving were used to keep mice. The environmental parameters in the animal room were: 50–60% humidity, 25°C temperature, and continuous ventilation.

### Melatonin and *Propionibacterium acnes*

Melatonin was purchased from Sigma Chemical (St. Louis, MO, USA). *P*. *acnes* type strain ATCC 6919 (also known as *Corynebacterium acnes* (Gilchrist) Eberson, NCTC 737) were purchased from Microbiologics (USA) and were grown overnight in Reinforced Clostridial broth (Oxoid, Uk) at 37°C under anaerobic conditions using anaerobic jar containing AnaeroGen sachets (Oxoid, UK). Single colonies were obtained by streaking the bacteria on Reinforced Clostridial agar plates (Oxoid, UK) and incubated under anaerobic conditions for 48 h. Few colonies were transferred from agar plates to Reinforced Clostridial broth and incubated anaerobically for 48 h to reach a density of 10^9^ cells/ml. Viable bacterial suspension was centrifuged and washed with PBS for intra-tumor injection.

### Cell line, culture conditions, and tumor inoculation

EMT6/P mouse breast carcinoma cells were purchased from Public Health England, catalogue No.: 96042344 (Salisbury, UK,). These cells were maintained using MEM supplemented with 10% FBS, 29 μg/ml L-glutamine, 40 μg/ml gentamicin and 1% penicillin-streptomycin. Tissue culture media and supplements were purchased from Sigma Chemical (St. Louis, MO, USA). Cells were harvested using trypsin-EDTA solution, centrifuged, washed, and re-suspended in PBS at a density of 5 X 10^6^ cells/ml. Trypan blue exclusion method was used to assess cell viability. Each mouse was subcutaneously injected in the abdominal area with 5 X 10^5^ cells suspended in 100 μl PBS.

### Anti-tumor activity

Sixteen days after tumor inoculation, mice were divided into four groups (N = 10 for each group) so that the average volume of tumors was closely matched for all groups. Group 1 served as a negative control and received daily intraperitoneal injection of PBS. Group 2 received a single intratumor injection of 1 X 10^6^ viable cells of *P*. *acnes* at day 1 of the treatment. Group 3 was exposed to two daily intraperitoneal injections of 10mg/kg melatonin (in 100 μl PBS) for 14 days. Melatonin was administered every 12 hours. Group 4 received a combination therapy of 1 X 10^6^ cells of *P*. *acnes* at day 1 and daily melatonin injections. Mice were observed daily during the treatment period (14 days) and the tumor size was measured using the equation: Length X width^2^ X 0.5 [22]. At day 14 of the treatment, mice were sacrificed (by cervical dislocation) and their tumor, lungs, and livers were isolated and stored in 10% buffered formalin.

### Histological examination of tumor, liver, and lung sections

Fixed organs were exposed to gradual dehydration using serial ethanol concentrations. Xylene was used to clear organs followed by wax infiltration using tissue processor (Thermo Shandon, UK). Rotary microtome (Reichert, Germany) was used to prepare 4 μm thick paraffin sections. Standard hematoxylin-eosin procedure was used to stain sections of different organs. Five samples from each group were examined to get a representative response.

### Immunohistochemical staining for VEGF

Tumor paraffin sections (4 μm thick) were deparaffinized by xylene followed by rehydration using serial ethanol concentration. Sections were immersed and heated (90°C) for 20 min in 10 mM citrate buffer (pH 6.0) to retrieve antigens. Inactivation of endogenous peroxidase activity was performed using 3% H_2_O_2_. Bovine serum albumin (5%) was used to block non-specific binding. Sections were then incubated with anti-VEGF antibodies at 4°C (Sigma, USA) overnight, followed by secondary antibody conjugated to horseradish peroxidase (Sigma, USA) for one hour. Visualization of antibodies was performed using 3,3'-diaminobenzidine solution (DAB) (Sigma, USA). Meyer's hematoxylin was used as a counter stain followed by dehydration and covering slides for microscopic examination.

### Detection of apoptosis in tumor sections

Terminal deoxynucleotidyl transferase (TdT) mediated-16-deoxyuridine triphosphate (dUTP) Nick-End Labelling (TUNEL) system (Promega, USA) was used to detect apoptosis in tissue sections. Tumor sections were de-paraffinized by xylene, washed in PBS, then rehydrated using serial concentrations of ethanol. Paraformaldehyde (4%) was used to fixed sections followed by washing in PBS and permeabilization using 20 μg/ml Proteinase K solution. Fragmented DNA was labeled by incubating sections with biotinylated dUTP in rTdT reaction mixture at 37°C for one hour and endogenous peroxidases were blocked using 0.3% hydrogen peroxide. Sections were incubated with streptavidin conjugated HRP at room temperature for 30 min. visualization of fragmented DNA was performed using hydrogen peroxide Followed by chromagen diaminobenzidine.

### IL-4 and INF-γ ELISA

Serum samples were prepared from fresh blood samples. Detection of IL-4 and IFN-γ was performed using commercially available ELISA kits according to the manufacturer instructions (Quantikine ELISA, R&D systems, USA).

### Assessment of liver functions

Commercially available kits (BioSystems, Spain).were used to quantitatively determine the serum levels of alanine transaminase (ALT) and aspartate transaminase (AST) as previously described [23].

### Statistical analysis

One-way analysis of variance (ANOVA) followed by *t*-test was used to measure variation between different groups. Significant difference between different groups was set at *P <* 0.05.

## Results

Treatment of tumor bearing mice with 10 mg/kg melatonin caused significant (P< 0.05) reduction in the average tumor size (-10.71%) compared with untreated tumor bearing mice that showed a huge increase in their tumors (96.79%) (Table 1).

**Table 1:** Effect of different treatments on tumor size and mortality.

Treatment	Initial tumor size (mm^3^)	% change in body weight	Final tumor size (mm^3^)	% change in tumor size	% death	% of cured mice
*Propionibacterium acnes*	308.17 ± 23.31	453.28 ± 16.15	47.08	0	10	+ 6.24
Melatonin	325.34 ± 19.41	290.48 ± 25.22	-10.71	20	0	-1.84
*Propionibacterium acnes* + Melatonin	323.19 ± 41.47	172.83± 31.85	-46.52	40	0	-2.95
Vehicle	313.39 ± 15.98	616.72 ± 44.36	96.79	0	20	+ 11.50

Ten mice were used in each group (N = 10).

On the other hand, although *P*. *acnes* treatment fails to reduce tumor growth; the overall increase in tumor size for this treatment was 47.08% which is significantly (P< 0.05) lower than the increase observed in the negative control (96.79%). The greatest reduction in the average tumor size was observed in the group treated with a combination therapy consisting of 10 mg/kg melatonin and intra-tumor injection of *P*. *acnes* with a decrease in average tumor size of -46.52% which is significantly higher than the decrease reported for melatonin (-10.71%) (Table 1). Measuring the change in the body weight of different treatments showed an increase (+11.5%) in the average body weight of the negative control group. Limited increase (+6.24) in the average body weight was observed in the group treated with *P*. *acnes* and a reduction in the body weight was detected in the groups treated with melatonin and combination therapies with average body weight of (- 1.84) and (- 2.95) respectively (Table 1). All mice exhibited normal activity throughout the study. However, mice that received melatonin therapy tend to cluster with reduced activity during the first 2 hours after melatonin injections.

**Fig 1:**
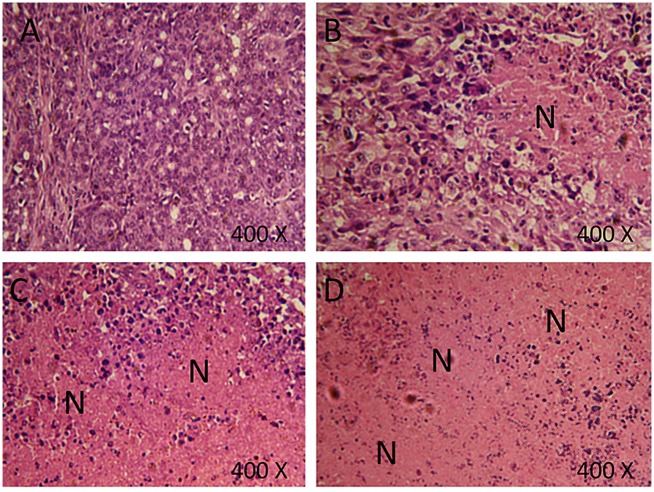
Hematoxylin/eosin staining of tumors treated with vehicle (A), 10mg/kg Melatonin (B), *Propionibacterium acne*s (C), and a combination of Melatonin and *Propionibacterium acne*s (D). N: Necrotic area. Extensive necrosis was evident in tumors treated with a combination of Melatonin and *Propionibacterium acnes*. Five mice were examined for each treatment.

For a better understanding of the observed effects, hematoxylin/ eosin staining was used to stain tumors of similar sizes from all groups. Large necrotic areas were detected in the tumors treated with melatonin compared with the negative control group. However, these areas are smaller than the necrotic regions observed in *P*. *acnes* single treatment (Fig 1). Extensive necrosis was observed in tumors treated with the combination therapy (melatonin + *P*. *acnes*). Larger and more frequent necrotic regions were detected in this treatment (Fig 1).

**Fig 2:**
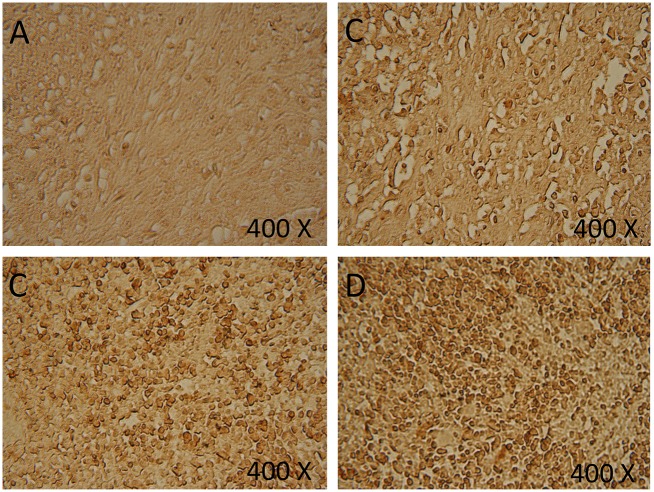
Tumor sections assayed by DeadEnd colorimetric TUNEL system to indicate cell apoptosis. (**A**) Negative control; (**B**) tumors treated with10mg/kg Melatonin; (**C**) tumors treated with *Propionibacterium acne*s; (**D**) tumors treated with a combination of Melatonin and *Propionibacterium acne*s. Brown stained nuclei indicate DNA fragmentation and nuclear condensation. Tumors of five mice for each treatment were examined to detect apoptosis.

In order to evaluate the ability of our combination treatment to induce apoptosis *in vivo*, tumors of different treatments were stained using TUNEL colorimetric assay. This assay detects DNA fragmentation resulted from programmed cell death. Treatment with melatonin caused an increase in the number of cells undergoing apoptosis compared with the negative control (Fig 2).

Similar results were obtained in tumors treated with *P*. *acnes* where high percentages of apoptotic cells were detected. However, the combination therapy (melatonin + *P*. *acnes*) caused higher percentage of apoptosis compared with single agent therapy (melatonin + *P*. *acnes*) (Fig 2).

**Fig 3:**
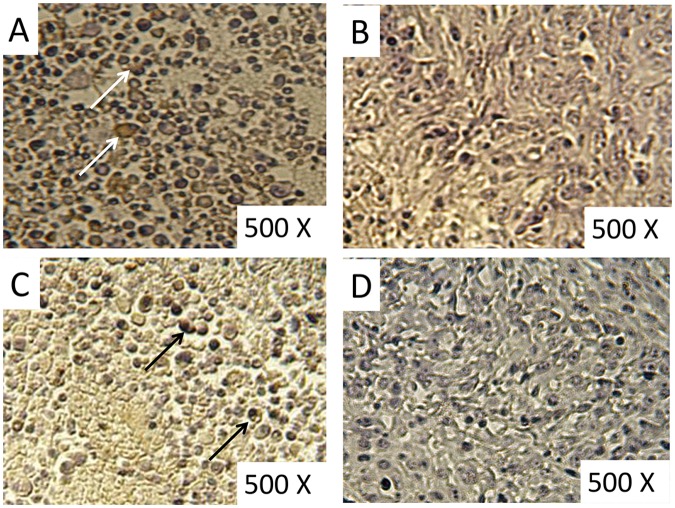
Immunohistochemistry staining of VEGF in tumor sections. (**A**) Negative control; (**B**) tumors treated with10mg/kg Melatonin; (**C**) tumors treated with *Propionibacterium acne*s; (**D**) tumors treated with a combination of Melatonin and *Propionibacterium acne*s. Yellow to brown stained cytoplasm indicates VEGF expression. Arrows shows tumor cells with stained cytoplasm. Tumors of 5 mice were stained for each treatment to detect VEGF expression.

To test whether angiogenesis inhibition may participates in the observed antitumor effect, VEGF expression was measures in tumor sections. VEGF was barely detectable in tumor sections treated with melatonin compared with the negative control (Fig 3).

The same result was obtained in tumors treated with the combination therapy (melatonin + *P*. *acnes*). On the other hand, *P*. *acnes* had little inhibitory effect against the expression of VEGF which was detected in high levels in tumor sections treated with this bacterium (Fig 3C).

**Fig 4:**
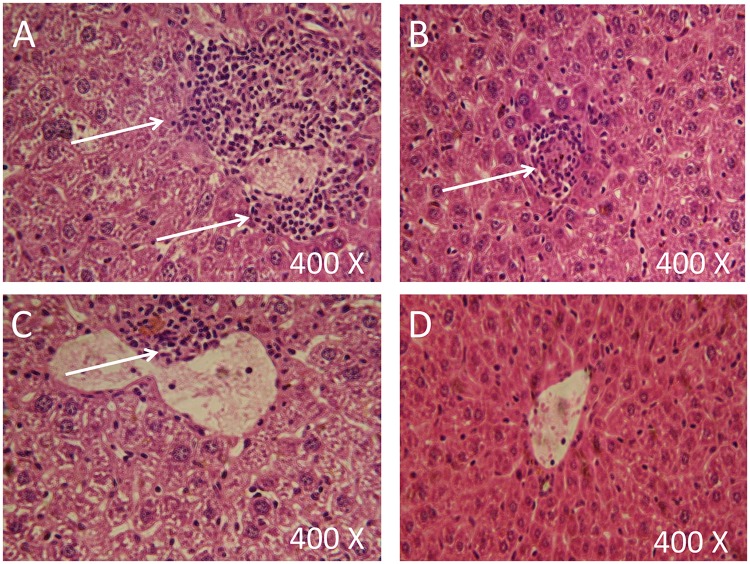
Breast cancer liver metastasis. (**A**) Negative control; (**B**) livers treated with10mg/kg Melatonin; (**C**) livers treated with *Propionibacterium acne*s; (**D**) livers treated with a combination of Melatonin and *Propionibacterium acne*s. Livers from five mice for each treatment were examined. Arrows point breast cancer cells between hepatocytes (cells in the background).

To test the ability of our combination to inhibit metastasis, sections from livers, lungs, and kidneys were prepared and stained using standard hematoxylin/ eosin procedure. Metastasis was not detected in lungs and kidneys samples (results not shown). However, a detectable metastasis was observed in liver sections. Breast cancer liver metastasis was lower in groups treated with melatonin or *P*. *acnes* compared with the negative control which has large areas of metastasis (Fig 4). On the other hand, the combination therapy (melatonin + *P*. *acnes*) was able to inhibit metastasis in liver. In this treatment, breast cancer cells were barely detected in liver sections.

To correlate between the histopathological results of the liver and liver functions, serum levels of Alanine transaminase (ALT) and aspartate transaminase (AST) were measured for all treatments. High levels of ALT and AST were detected in serum samples collected from untreated tumor bearing mice with AST and ALT values of 350.62 and 185.10 IU/L, respectively (Table 2). These values are significantly higher than the values of these enzymes in healthy mice that showed concentrations of 37.55 and 14.08 IU/L for AST and ALT, respectively. Compared with the untreated group, treatment of tumor bearing mice with *P*. *acnes* significantly (P< 0.05) reduced the levels of AST and ALT to 117.55 and 101.50 IU/L, respectively. Further reduction was detected in the group treated with melatonin with AST and ALT values of 110.87 and 87.08 IU/L, respectively. Although the combination therapy (melatonin + *P*. *acnes*) did not cause a reduction in the concentrations of liver enzymes to their normal levels, this treatment produced the lowest levels of these enzymes compared with other treatments with AST and ALT levels of 66.32 and 23.25 IU/L respectively.

**Table 2:** Effect of different treatments on serum levels of liver enzymes in tumor bearing mice (N = 5).

Treatment	AST (IU/L) ± SEM	AST (IU/L) ± SEM
*Propionibacterium acnes*	117.55 ± 9.53	101.50 ± 5.60
Melatonin	110.87 ± 12.33	87.08 ± 7.89
*Propionibacterium acnes* + Melatonin	66.32 ± 5.02	23.25 ± 4.32
vehicle	350.62 ± 13.57	185.10 ± 15.24
Normal control (mice without tumors)	37.55 ± 3.45	14.08 ± 3.60

Data are expressed as the mean ± SEM of 5 mice.

In order to explore the changes in the immune response associated with each treatment, serum levels of IL-4 and IFN-γ were detected using ELISA kits. Our results suggest that mice treated with melatonin, *P*. *acnes*, and the combination therapy had high levels of IFN-γ compared with the negative control group. On the other hand, no significant change in the levels of IL-4 was detected in these treatments. Additionally, mice treated with the combination therapy had significantly higher IFN-γ compared with groups that received single therapy (Table 3).

**Table 3:** Serum levels of INF-γ and IL-4 for different treatments.

	INF-γ (pg/ml)	IL-4 (pg/ml)
*Propionibacterium acnes*	34.12 ± 3.34	4.12 ± 1.65
Melatonin	128.08 ± 5.20	4.31± 1.07
*Propionibacterium acnes* + Melatonin	142.67 ± 4.35	3.52 ± 1.40
vehicle	17.03 ± 2.17	3.63 ± 0.67

Data are expressed as the mean ± SEM of 5 mice (p-value < 0.05).

## Discussion

In the present study the antitumor effect of a combination of melatonin and *P*. *acnes* was evaluated against breast cancer inoculated in mice. This combination therapy showed high ability to control breast cancer progression *in vivo* by mechanisms involving apoptosis induction, angiogenesis inhibition, and stimulation of Th1 immune response.

The use of bacteria in cancer therapy was reported in previous studies that showed the anticancer and immune-modulatory activities of different bacterial strains including *Clostridium novyi* [24], *Bifidobacterium longum* [25], *Salmonella typhi* [26], and *P*. *acnes* [18].

In our study *P*. *acnes* reduced the growth rate of breast cancer. This result agrees with the previous findings that reported the antitumor activity of *P*. *acnes* against different mouse cell lines including mastocytoma, fibrosarcoma, and mammary carcinoma [12].Mouse lung carcinoma and brain tumors were also inhibited by *P*. *acnes* [27, 28]. Our results showed that the anticancer activity of *P*. *acnes* is mediated by inducing apoptosis and stimulating INF-γ production which is a key cytokine in Th1 antitumor immune response. Additionally, this treatment slightly reduced the percentage death to 10% compared with 20% in the control group and produced large necrotic regions in tumor core. Our results are consistent with the previous findings that reported a potent antitumor and imunomodulatory effect of *P*. *acnes* against malignant melanoma [18] and other cancers [12],. However, the levels of INF-γ induced by *P*. *acnes* are relatively low if compared with the levels reported by previous studies. This difference is mainly due to the difference in the strain of *P*. *acnes* used in anticancer therapy. Previous studies showed that using different strains of *P*. *acnes* can induce different immune responses including enhanced Fc expression on macrophages [29], activation of antitumor T-cells [30], and stimulating neutrophil activity [16]. Additionally, failure of *P*. *acnes* to induce an immune response was also reported [19]. The results of a more recent research showed that *P*. *acnes* activation of the immune system is mediated by both TLR9 dependent and independent pathways. Furthermore, only *P*. *acnes* strains that resist cellular digestion can persist for longer time *in vivo* and stimulate the immune system [31]. In spite of the potent anticancer effects of *P*. *acnes*, this bacterium did not cause significant inhibition in the expression of VEGF which is one of the main mediators in the angiogenesis process. Also, this bacterial treatment did not inhibit cancer metastasis to the liver. This result is in contrary with the previous findings that showed anti-metastasis effect of this bacterium against mouse lung carcinoma [28]. and Lewis tumour pulmonary metastasis [32].

In addition to the strain used, the efficiency of *P*. *acnes* antitumor activity depends on many factors including type of the targeted tumor, tumor immunogenicity, tumor size and location, the dose of bacteria injected, and the route of injection [12]. In our study the tumor model was mouse breast carcinoma (EMT6/P) which is one of the highly metastatic cancers and was resistant to *P*. *acnes* anti-metastasis effect.

The effect of *P*. *acnes* is not limited to cancer cells. Previous studies showed the ability of this bacterium to interfere with the function of normal cells. Treatment of non-malignant prostate epithelial cells with *P*. *acnes* induced the production of IL-6, IL-8, and GM-CSF [33].These cytokines are essential for the attraction and differentiation of neutrophils and macrophages in the site of inflammation [34].Although such inflammatory response is part of the antitumor effect of this bacterium (as indicated by our results), previous studies showed that both IL-6 and IL-8 may contribute to the development of prostate cancer by stimulating cell proliferation [35, 36].Difference in virulence traits were observed in different *P*. *acnes* subpopulations [37].Previous studies reported a genetic and biochemical differences between *P*. *acnes* isolated from skin and that isolated from prostate [38].Both isolates were able to induce inflammatory response. However prostate-derived strain persists for longer time (3 months after infection) [39] which may explain its ability to induce prostate cancer. The strain of *P*. *acnes* used in our study was derived from skin and is less expected to cause prostate cancer.

Angiogenesis inhibition is an attractive target in cancer prevention and therapy as it deprives tumor of oxygen and nutrients which reduces tumor proliferation and expansion [40]. However, anti-angiogenic therapy alone cannot cause complete tumors regression because it acts indirectly on cancer cells [41].

In our attempts to augment the antitumor effect of *P*. *acnes*, melatonin was combined with the bacterial therapy as an antiangiogenic agent. Such combination therapy may increase the anticancer therapeutic stress without altering the antitumor activity of *P*. *acnes* which can resist low oxygen concentrations. In this context, melatonin single treatment caused an improvement in tumor size regression. This regression was associated with apoptosis induction, increased INF-γ production, low expression of VEGF, and a decrease in percentage death to 0%. Additionally, small areas of necrosis were observed in tumor core. These effects of melatonin were reported in previous studies that showed the ability of this hormone to cause a reduction in tumor growth, cell proliferation, and an inhibition of angiogenesis in breast cancer cells inoculated in mice [8].On the other hand, melatonin single therapy failed to prevent cancer metastasis and cancer cells were detected in liver sections treated with melatonin.

This is the first study to evaluate the anticancer therapeutic potential of a combination consisting melatonin and *P*. *acnes*. This combination worked synergistically to cause the highest regression in tumor size with geographical necrosis in the tumor core and enhanced apoptosis. Moreover, the expression of INF-γ was stimulated and VEGF expression was inhibited in tumor bearing mice treated with this combination. Additionally, this combination exhibited high potential to reduce cancer metastasis which was detected in liver samples of other groups. While, lungs and kidneys of all treatments were free of breast cancer cells.

A possible explanation for the liver metastasis is the dense vasculature (branches of veins and arteries) and the arrangement of liver cell plates into two rows of hepatocytes with direct association with sinusoidal blood. These histologic properties of the liver reduce the chance of developing chronic and acute hypoxia and provide oxygen rich environment for cancer survival [42].The histology of other tissues provide less encouraging environment for cancer metastasis.

Cancer metastasis to the liver can be effectively evaluated by measuring liver enzymes such as AST and ALT that reflects the normal function of the liver [43]. In order to evaluate the extent of hepatic metastasis, serum levels of AST and ALT were measured for all treatments. High levels of both enzymes were detected in all treatments. However, the combination therapy caused a reduction in the levels of both enzymes to concentrations closer to their normal values compared with other treatments.

In our study, all groups treated with melatonin showed high levels of INF-γ and normal levels of IL-4. Healthy individuals have a balanced ratio of Th1/Th2 cytokines. Increased concentrations of Th2 cytokines were observed in patients harboring different tumor types [44]. T helper cells express G-protein-coupled melatonin cell membrane receptors. Binding of melatonin to these receptors stimulates the production of Th1 cytokines including INF-γ [45]. On the other hand, melatonin has no effects on the levels of IL-4 which is a Th2 cytokine. Furthermore, production of IL-1, IL-6, and IL-12 was also stimulated by melatonin [46]. Inhibition of Th2 cytokines is mediated by high levels of INF-γ which was observed in groups treated with melatonin [47]. Additionally, this Th1 cytokine has an important role in regulating antitumor immune response [48].

In conclusion, a combination of melatonin and *P*. *acnes* can work synergistically (or additively) to inhibit breast cancer inoculated in mice. *P*. *acnes* antitumor effect is mediated by apoptosis induction and immune system stimulation. Melatonin augments *P*. *acnes* anticancer effects by angiogenesis inhibition, apoptosis induction, and stimulation of the immune system. This combination is suitable for further investigations to be considered as a possible therapeutic option to be used in clinical trials. However, measurement of other immune mediators (IL-12 and TNF-α) can provide a better understanding of the immune response obtained by this combination therapy.

## Supporting Information

S1 Table Melatonin and *Propionibacterium acnes* anticancer mechanisms. Summary of the anticancer activity mechanisms mediated by melatonin, *Propionibacterium acnes*, and their combination. (+: active, ++: highly active,—: no activity, -/+: slight activity).
(PDF)
