# Supplementary material for: Propionibacterium acnes Augments Antitumor, Anti-Angiogenesis and Immunomodulatory Effects of Melatonin on Breast Cancer Implanted in Mice
Source: PLoS One. 2015 Apr 28;10(4):e0124384. doi: 10.1371/journal.pone.0124384 (PMC4412818; doi:10.1371/journal.pone.0124384)
Supplement: S1 Table — Summary of the anticancer activity mechanisms mediated by melatonin, Propionibacterium acnes, and their combination. (+: active, ++: highly active,—: no activity, -/+: slight activity). (PDF) [file pone.0124384.s001.pdf]

S1 Table:

Summary of the anticancer activity mechanisms mediated by melatonin, *Propionibacterium acnes*, and their combination. (+: active, ++: highly active, - : no activity, -/+: slight activity).

**Anticancer mechanism**	**Melatonin**	***Propionibacterium acnes***	**Combination effect**
Apoptosis induction	+	++	+++
Inhibition of VEGF expression	+	-	+
INF-γ induction	++	+	+++
Induce tumor necrosis	+	++	+++
Reduction of tumor size	+	-	++
Inhibit metastasis	-/+	-/+	+
